# Formation of Humic Substances in Weathered MSWI Bottom Ash

**DOI:** 10.1155/2013/384806

**Published:** 2013-06-05

**Authors:** Haixia Zhang, Takayuki Shimaoka

**Affiliations:** ^1^School of Urban Construction, Hebei University of Engineering, Handan 056038, China; ^2^Department of Urban and Environmental Engineering, Kyushu University, Fukuoka 819-0395, Japan

## Abstract

The study aimed at evaluating the humic substances (HSs) content from municipal solid waste incinerator (MSWI) bottom ash and its variation with time and the effect of temperature on HSs formation. The process suggested by IHSS was applied to extract HSs from two different bottom ash samples, and the extracted efficiency with NaOH and Na_4_P_2_O_7_ was compared. MSWI bottom ash samples were incubated at 37°C and 50°C for 1 year. HSs and nonhumic substances were extracted from the bottom ash sample with different incubated period by 0.1 M NaOH/Na_4_P_2_O_7_. Results show that the rate of humic acid formation increased originally with incubation time, reached a maximum at 12th week under 37°C and at 18th week under 50°C, and then decreased with time. More humic acid in MSWI bottom ash was formed under 50°C incubated condition compared with that incubated under 37°C. Also, the elemental compositions of HSs extracted from bottom ash are reported.

## 1. Introduction

Incineration process displays an important role in the municipal solid waste (MSW) management in Japan. In the recent years, approximately 78% by weight of MSW is incinerated. Incineration is a multipurpose strategy aiming at fighting pollution of environment, energy saving, maximizing benefits of waste, mineralizing and stabilizing waste, and reducing the volume of waste. However, in practice, organic matters in MSW are not completely oxidized by incineration. A few percentage of partially unburned organic waste materials still exist with subsequent formation of new organic compounds being left behind forming the bottom ash [1–5]. European countries restrict the organic matter content in the landfill waste to 5% in the aim to reduce the pollution from landfill waste [6]. Several researches showed that dissolved organic matter (DOM) in MSWI bottom ash leachate contributes to Cu leaching [7, 8]. The DOM of MSWI bottom ash also would lead to carbonization and to pH value decrease in landfill site [2].

Organic matter in MSWI bottom ash is divided into humic substances (HSs, mainly composed of humic acid and fulvic acid) and nonhumic substances (NHS), based on the soil organic matter classification. HSs widely exist in the natural environment and have a high affinity for binding heavy metals and organic pollutants. Humic acid and fulvic acid have been identified as important DOC subfractions contributing to the complexation of contaminants [9]. The study by Zomeren and Comans [8] found that humic acid and fulvic acid contributed to 0.3–0.6% and 14–26% DOC, respectively, in the leachate from MSWI bottom ash. Their study further suggests that the fulvic acid-type components exist in MSWI bottom ash leachates are likely responsible for Cu leaching from bottom ash [10]. The study by Kim revealed that the leaching concentration of dioxins increased with advanced humicification and showed relatively good correlation to DOC [11]. However, there was little information about the formation and decomposition of HSs in MSWI bottom ash.

This work compared the efficiency of NaOH and Na_4_P_2_O_7_ reagents on the extraction HSs from MSWI bottom ash and the effect of temperature on the formation of humic acid from the incineration of the MSW bottom ash. A lab-scale incubation facility was used to help exploration of the humic acid formation mechanism.

## 2. Materials and Methods

### 2.1. Materials and Sampling

The fresh quenched MSWI bottom ashes were taken from O-incineration plant and R-incineration plant, named as O-BA and R-BA. O-incineration plant was operated on a semicontinuous type and incinerated at 850°C with a daily treatment capacity of 130 t/d (65 t/16 h × 2). R-incineration plant applied stoker furnace operated on a continuous type, with an incineration capacity of 900 t/d (300 t × 3) and the temperature in the combustion chamber was 900°C. The two bottom ash samples and a crushed incombustibles sample were passed through 4.75 mm sieve.

### 2.2. Extraction of Humic Substances

**Figure 1:**
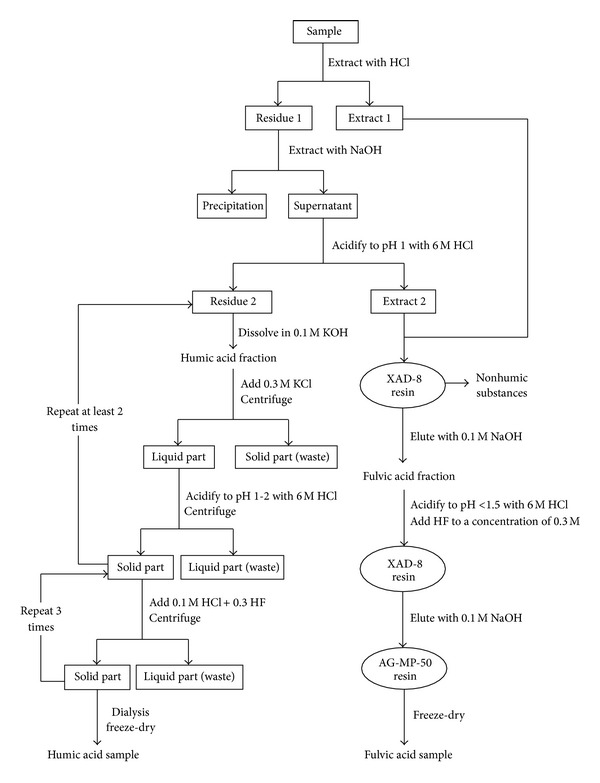
Schematic diagram of fractionation of humic substances and nonhumic substances.

Organic matter in MSWI bottom ash contains humic and fulvic acids together with other nonhumin substances. Humic acid and fulvic acid were separated from solid samples applying the method of international humic substances society (IHSS).  Figure 1 shows the conceptual steps of the separation technology.

**Step 1 (extraction with HCl)**

Distilled water was added to the sample in the container to maintain L/S ratio < 10. Aqueous fluid is then acidified using HCl to a pH value of 1-2. Adjust the L/S ratio to 10, and the container was closed for shaking for 1 hour. The container was then centrifuged followed by filtration using filter paper to attain residue 1 and extract 1. This step is helpful to remove part of carbonate in MSWI bottom ash.

**Step 2 (extraction with NaOH)**

Neutralize the residue 1 using NaOH to pH 7 followed by dilution with 0.1 M NaOH under an atmosphere of N_2_ to attain L/S of 10. Shake the solution under atmosphere of N_2_ for 4 hours. Supernatant was separated from the precipitate by centrifugation and filtration. The supernatant was acidified to pH 1 with HCl and then centrifuged to obtain extract 2 and residue 2.

In this step, Na_4_P_2_O_7_ or mixture of NaOH/Na_4_P_2_O_7_ was also applied instead of NaOH.

**Step 3 (purification of humic acid fraction)**

Dissolve the residue 2 using 0.1 M KOH under N_2_ to obtain humic acid fraction. Add 0.3 M KCl solution to humic acid fraction to reach L/S 10. Aqueous fluid was separated from the solid resides by centrifugation and filtration to obtain solid part and liquid part. Waste the solid part and liquid part was acidified to pH 1-2 using HCl and then centrifuged. Solid part was separated by centrifugation. Repeat the process previously mentioned at least 2 times.

Sequentially, dissolve the solid part using 0.1 M HCl/0.3 M hydrofluoric acid (HF) solution in a plastic container. Shake the solution for 24 hours. Solid part was separated from the liquid part by centrifugation and filtration. Repeat the process previously mentioned 3 times. Transfer the solid part to a dialysis tube by slurring with water and dialyze against distilled water until the dialysis water gives a negative Cl^−^ test with the AgNO_3_. At last, the solid part from dialysis tube was freeze-dried to obtain the solid humic acid sample.

**Step 4 (purification of fulvic acid fraction)**

NHS was recovered from the solutions obtained from Steps 1 and 2 applying resin exchange technique. The resin used was XAD-8, ion-exchange resin.

Elute the column with 0.1 M NaOH. Pass eluate through H^+^-saturated cation exchange resin (AG-MP-50). Freeze-dry the eluate to recover the H^+^-saturated fulvic acid.

The concentrations of humic acid fraction, fulvic acid fraction, and NHS fraction in carbon were determined with a TOC analyzer (TOC-V, Shimadzu Co.).

### 2.3. Incubation Experiment

The pretreatment R-BA was prepared for the incubation experiments. About 4 kg R-BA was filled in each stainless steel container with untight covers. The containers were put in constant temperature ovens setting at 37°C and 50°C, respectively. The incubation experiments were continued for one year under aerobic conditions. During the incubation process, the water contents of the samples were adjusted to 30% by adding distilled water every day, simultaneously mixing the samples.

Approximately 200 g samples were taken out from the containers after 2, 4, 8, 12, 18, 24, 32, 36, 44, and 52 weeks of incubation period. These samples were air-dried, grinded, and sieved through a 2 mm mesh. The pretreated samples were kept in closed plastic bags and saved under 4°C till analysis.

### 2.4. Elementary Analysis of Humic Substances

Carbon, hydrogen, and nitrogen contents in eluted humic, fulvic acid were determined with the help an elemental analyzer (YANAKO CHN coder MT-50, Yanagimoto Co.).

## 3. Results and Discussion

### 3.1. Effect of Extraction Reagent on Humic Substances Extraction

**Figure 2:**
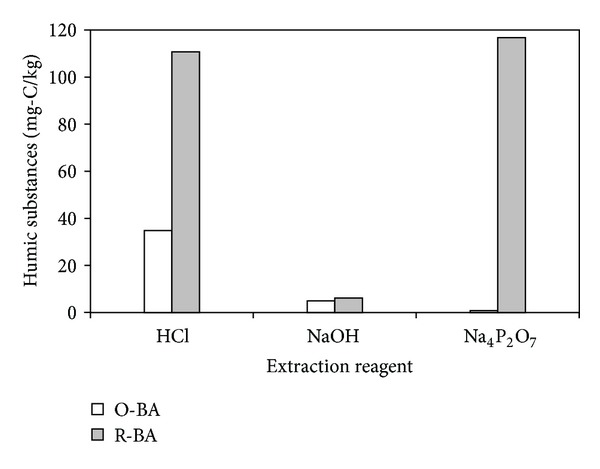
Amount of humic substances extracted from R-BA and O-BA by HCl, NaOH, and Na_4_P_2_O_7_ in sequence.

The amounts of HSs extracted from O-BA and R-BA by three extraction reagents are shown in Figure 2. HSs extracted by HCl, NaOH, and Na_4_P_2_O_7_ from O-BA accounted for 86%, 12%, and 2% of total HSs amount, while these values were 47%, 3%, and 50% for R-BA. The data indicated that HCl was effective to extract HSs from both MSWI bottom ash samples and NaOH had minor effect on extraction of HSs from the two samples. Na_4_P_2_O_7_ almost could not extract HSs from O-BA, but it was an effective reagent to extract HSs from R-BA.

IHSS has been suggested that the material should be dissolved in dilute HCl before the alkaline extractant is applied, which is helpful to remove Ca and other polyvalent cations and increases the efficiency of extraction of organic matter with alkaline reagents. The previous study has indicated that the main minerals in MSWI bottom ash contain Ca and other polyvalent cations (such as Fe and Al) [12]. So HCl is a necessary reagent to extract HSs from MSWI bottom ash. NaOH has been expected to extract mainly free complexed organic components and Na_4_P_2_O_7_ to extract intimately complexed organic part, which can form complexes with exchangeable polyvalent cations, thereby breaking down the cation bridges between the exchangeable cations and organic matter [13, 14]. According to the following reactions postulated by Alexsandrova [15], Na_4_P_2_O_7_ are thought to extract OM by breaking down the cation bridges between Ca and other polyvalent cations and organic matter and forming insoluble precipitates:

(1) R(COO)4Ca2+Na4P2O7→R(COONa)4+Ca2P2O7↓2[RCOOX(OH)2](COO)2Ca+Na4P2O7  →2[RCOOX(OH)2](COONa)2+Ca2P2O7↓

According to the experiment results, it could be expected that R-BA contain less free complexed organic matter and considerable amount of complexed organic matter. For the purpose of investigating the HSs content in MSWI bottom ash, here it is suggested to combine NaOH and Na_4_P_2_O_7_ to extract HSs from MSWI bottom ash, following HCl extraction. 

The total HSs amounts extracted from the two bottom ash samples differed to a large extent. O-BA only contained 40.5 mg-C/kg HSs, but R-BA contained 234 mg-C/kg HSs which was almost 6 times higher than that of O-BA. This may be caused by the fact of the different composition of MSW treated in the two incineration plants. In addition, the different incinerator type and incineration temperature can also have an effect on the organic components in the MSWI bottom ash.

### 3.2. Effect of Temperature on the Humic Substances Formation

**Figure 3:**
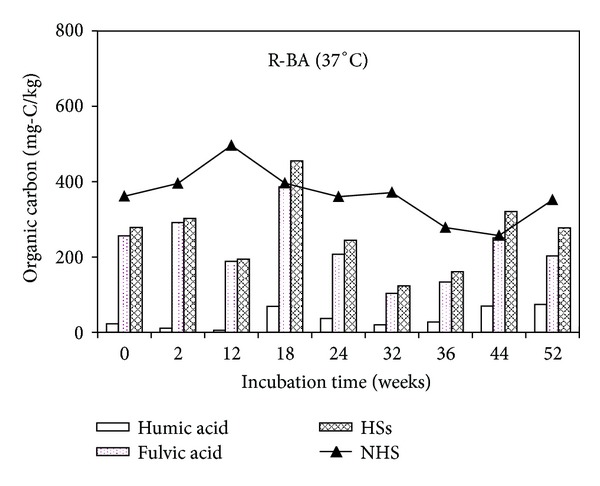
Humic substances and nonhumic substances contents as a function of incubation time in R-BA incubated under 37°C.

Figure 3 shows the content of humic acid, fulvic acid content as well as HSs in the R-BA incubated under 37°C. Fresh R-BA contained 279 mg-C/kg HSs (23 mg-C/kg humic acid and 256 mg-C/kg fulvic acid) and 361 mg-C/kg NHS. Thus it can be seen that fresh MSWI bottom ash contained HSs after experiencing an incineration process. In the case of the bottom ash samples incubated under 37°C, humic acid concentration was in the range of 6–74 mg-C/kg and fulvic acid in the range of 103–286 mg-C/kg through the incubation period. The NHS content in R-BA increased at the initial incubation stage, and it reached 496 mg-C/kg at 12th weeks then it began to decrease.

**Figure 4:**
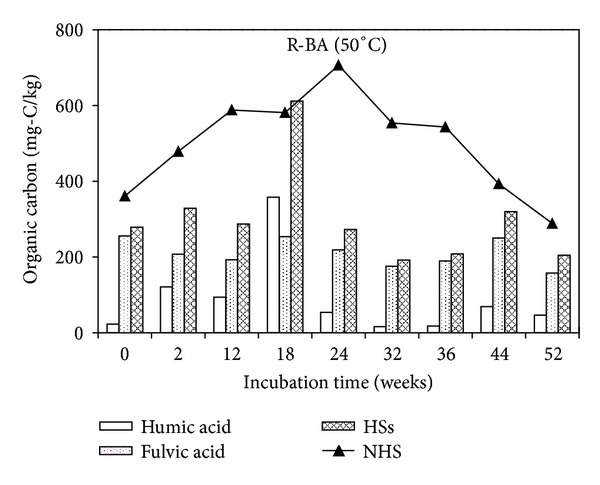
Humic substances and nonhumic substances contents as a function of incubation time in R-BA incubated under 50°C.

For the R-BA samples incubated under 50°C shown in Figure 4, humic acid was in the range of 16–358 mg-C/kg, and fulvic acid in the range of 158–256 mg-C/kg. Humic acid was produced rapidly under 50°C until 18th week and the amount of humic acid once exceeded that of fulvic acid at 18th week, and then humic acid content dropped distinctly. Fulvic acid content was relatively stable through the incubation period. Humic acid mainly comes from the degradation of lignin, and higher temperature was good for lignin degradation [16]. Compared with R-BA incubated under 37°C, there is more humic acid contained in R-BA incubated under 50°C.

For MSWI bottom ash incubated under 50°C, the humic acid content was significantly higher than that of the MSWI bottom ash incubated under 37°C. Reversely, the highest content value of fulvic acid appeared in the sample incubated under 37°C, showing that high temperature may be beneficial to the formation of humic acid, while low temperatures are conducive to the accumulation of fulvic acid. The total content of humic acid and fulvic acid amount was higher in the sample incubated under 50°C compared with that under 37°C and so as NHS.

This shows that temperature is an important environmental factor of HSs formation and transformation. The storage temperature of MSWI bottom ash will affect the HSs content, thereby affecting the migration of heavy metals.

### 3.3. Elementary Composition of Humic Substances from MSWI Bottom Ash

The elementary compositions of humic acid and fulvic acid extracted from R-BA are shown in Table 1 together with the average values of soil HSs in [17]. Compared with the literature data, the content of carbon and hydrogen in HSs extracted from R-BA accorded with those of soil HSs. The extracted HSs from R-BA had a higher content of nitrogen and lower content of oxygen compared with the soil HSs. HSs decompose slowly and supply nitrogen nutrient to microbial utilization and release carbon dioxide. Simultaneously, the dead microorganism supplies carbon source for HSs synthesis. The loss of nitrogen is substantial compared to carbon. Nitrogen loss caused C/N ratio of MSWI bottom ash to gradually increase with incubation time.

## 4. Conclusions

The study determines the HSs extraction reagent from MSWI bottom ash by comparison of NaOH and Na_4_P_2_O_7_. NaOH and Na_4_P_2_O_7_ have different extraction efficiency for the studied two MSWI bottom ashes. So 0.1 M NaOH/0.1 M Na_4_P_2_O_7_ was recommend to extract HSs from MSWI bottom ash.

Fresh MSWI bottom ash contains humic acid and fulvic acid. More humic acid was formed during the incubation period and accounted for 8–27% of the organic fractions in the landfilled MSWI bottom ash. Fulvic acid was contained in the fresh MSWI bottom ash, and its amount was relatively stable. The variation of HSs content in the incubated samples showed a change with incubation time. The results showed that high temperature may be beneficial to the formation of humic acid, while low temperatures are conducive to the accumulation of fulvic acid.

## Figures and Tables

**Table 1:** Elementary analysis of humic substance extracted from R-BA.

	Sample	C (%)	H (%)	N (%)	O (%)	C/N
Humic acid	Fresh R-BA	53.68	5.63	6.52	34.17	8.23
	24th weeks incubated R-BA	54.81	5.00	5.55	34.64	9.88
	Tan, 2003 [17]	53.8–58.7	3.2–6.2	2.6–5.05	39.7–48.8	12.3–17.3

Fulvic acid	Fresh R-BA	46.5	4.34	1.78	47.38	26.12
	Tan, 2003 [17]	40.7–50.6	3.8–7.0	0.8–4.3	32.9–51.9	18.4–37.8
